# KBN2202, a Salicylic Acid Derivative, Preserves Neuronal Architecture, Enhances Neurogenesis, Attenuates Amyloid and Inflammatory Pathology, and Restores Recognition Memory in 5xFAD Mice at an Advanced Stage of AD Pathophysiology

**DOI:** 10.3390/ijms262210942

**Published:** 2025-11-12

**Authors:** Sun-Young Lee, Jong Chul Kim, Mi Ran Choi, Jiseo Song, Moonhang Kim, Seok-Hwan Chang, Jong Sung Kim, Joon-Suk Park, Sang-Rae Lee

**Affiliations:** 1 Efficacy Test Center for Mental & Behavioral Disorders (MBD), Ajou University Hospital, Suwon 16499, Republic of Korea; sun02lee@ajou.ac.kr (S.-Y.L.); mrchoi2007@ajou.ac.kr (M.R.C.); jsnim11@ajou.ac.kr (J.S.); mook1052@ajou.ac.kr (M.K.); tjrghksekrzj@ajou.ac.kr (S.-H.C.); kjs0829@ajou.ac.kr (J.S.K.); 2 Preclinical Research Center, Daegu-Gyeongbuk Medical Innovation Foundation (K-MEDI hub), Daegu 41061, Republic of Korea; kimjc7e@kmedihub.re.kr; 3 Laboratory Animal Research Center, Ajou University School of Medicine, Suwon 16499, Republic of Korea; 4 Department of Pharmacology, Ajou University School of Medicine, Suwon 16499, Republic of Korea; 5 BK21 R&E Initiative for Advanced Precision Medicine, Suwon 16499, Republic of Korea; 6 Department of Biomedical Sciences, Graduate School of Ajou University, Suwon 16499, Republic of Korea

**Keywords:** Alzheimer’s disease, KBN2202, 5xFAD mice, salicylic acid derivative, neurogenesis, neuroinflammation

## Abstract

Alzheimer’s disease (AD) is a progressive neurodegenerative disorder characterized by cognitive decline, amyloid-β (Aβ) pathology, synaptic degeneration, impaired neurogenesis, and chronic neuroinflammation. KBN2202, a small-molecule salicylic acid derivative [2-[(2-naphthalen-1-yloxy)ethyl]amino]-4-hydroxybenzoic acid], was investigated for its potential as a multi-target therapeutic agent in advanced-stage AD. To this end, 9-month-old 5xFAD mice with established AD-like pathology received daily oral KBN2202 (5 or 20 mg/kg) or vehicle for 12 weeks. KBN2202 demonstrated broad histopathological benefits. It preserved hippocampal CA1 cytoarchitecture and increased dendritic length in cortical neurons. Neurogenic activity was also enhanced, with elevated doublecortin (DCX) expression in the subventricular zone (SVZ). At the molecular level, KBN2202 reduced amyloid precursor protein C-terminal fragments (APP-CTFs), key intermediates in amyloidogenic processing, and histological staining confirmed a significant reduction in fibrillar and diffuse Aβ plaque burden in the cortex and hippocampus. Furthermore, KBN2202 attenuated astrocytic and microglial activation, indicating suppression of chronic neuroinflammation. In behavioral assessments, KBN2202 significantly improved recognition memory in the novel object recognition (NOR) test, while Y-maze performance remained unchanged. Overall, the compound exhibited robust neuroprotective, pro-neurogenic, anti-amyloid, and anti-inflammatory effects. These findings support the therapeutic potential of KBN2202 as a multi-functional candidate for symptomatic-stage AD.

## 1. Introduction

Alzheimer’s disease (AD) is a complex neurodegenerative disorder marked by progressive cognitive decline, extracellular amyloid-β (Aβ) deposition, synaptic and neuronal loss, tau pathology, and chronic neuroinflammation [1,2,3]. Among these pathological processes, neuroinflammation plays a pivotal role by disrupting synaptic integrity and suppressing neurogenic potential, thereby further accelerating cognitive impairment [4,5,6]. Collectively, these interconnected pathological processes underscore the need for therapeutic strategies that transcend amyloid clearance and instead provide broad, multi-level neuroprotection.

Despite extensive research efforts, most current AD therapies primarily target single pathological pathways, such as amyloid or tau, and have shown only modest symptomatic benefits without modifying disease progression [7,8,9]. Monoclonal antibodies against Aβ can reduce plaque burden but often fail to restore neuronal or cognitive function and may cause adverse vascular events [10,11]. Likewise, anti-inflammatory agents and neurotrophic modulators have yielded inconsistent outcomes, reflecting the multifactorial nature of AD pathogenesis [12,13,14]. Therefore, there remains an urgent need for therapeutics capable of simultaneously modulating multiple interconnected pathological processes [15,16,17].

Salicylic acid derivatives, beyond their well-known anti-inflammatory effects, have demonstrated direct neuroprotective actions, including mitigation of oxidative and excitotoxic neuronal injury [18,19]. For example, salicylic acid and its derivatives have been shown to protect dopaminergic neurons by reducing oxidative stress and abnormal dopamine metabolism in experimental models of Parkinson’s disease [20]. Aspirin and salicylate have also displayed neuroprotection in MPTP (1-methyl-4-phenyl-1,2,3,6-tetrahydropyridine)-induced dopaminergic injury models, likely via hydroxyl radical scavenging [21]. Recently, multifunctional tacrine–salicylamide conjugates showed antioxidant, anti-Aβ aggregation, and cholinesterase inhibitory activities, thereby supporting neuronal resilience [22].

KBN2202 is a small-molecule salicylic acid derivative [2-[(2-naphthalen-1-yloxy)ethyl]amino]-4-hydroxybenzoic acid] designed to integrate neuroprotective, anti-inflammatory, and neurogenic actions. In vitro studies have shown that KBN2202 suppresses the production of neuroinflammatory mediators such as nitric oxide and reactive oxygen species in lipopolysaccharide (LPS)-stimulated microglial cells [23]. Based on this pharmacological profile, we hypothesized that KBN2202 exerts broad therapeutic effects by concurrently promoting neuroprotection and neurogenesis, modulating amyloidogenic processing, and suppressing inflammation, rather than functioning solely as an anti-inflammatory compound. Furthermore, given its potential to preserve cortical and hippocampal integrity, we anticipated that KBN2202 would also improve cognitive performance. This study aimed to demonstrate that a rationally designed salicylic acid derivative can simultaneously modulate multiple AD-related mechanisms even at the symptomatic stage, thereby distinguishing it from conventional single-target therapies and underscoring its translational potential.

To test these hypotheses, we evaluated the therapeutic effects of KBN2202 in 9-month-old 5xFAD mice, which exhibit symptomatic AD-like pathology characterized by neuronal loss, dendritic degeneration, amyloid accumulation, gliosis, and cognitive impairment [24,25]. In addition to histopathological and molecular analyses, cognitive performance was assessed using the Y-maze and novel object recognition (NOR) tests. As treatment was initiated at a late disease stage, spatial working memory remained impaired, whereas recognition memory showed significant improvement.

This study was designed to comprehensively determine whether long-term oral administration of KBN2202 could attenuate amyloid pathology, neuroinflammation, and neurodegeneration while supporting neurogenesis and cognitive function. By integrating neuropathological and behavioral outcomes, we sought to determine whether KBN2202 confers broad neuroprotective benefits rather than isolated effects on a single pathway. Collectively, these findings indicate that KBN2202 alleviates amyloid, inflammatory, and neurodegenerative pathology while restoring recognition memory, thereby demonstrating both structural and functional efficacy in symptomatic-stage AD mice.

## 2. Results

### 2.1. Neuroprotective Effect of KBN2202 on Hippocampal CA1 Structure

**Figure 1:**
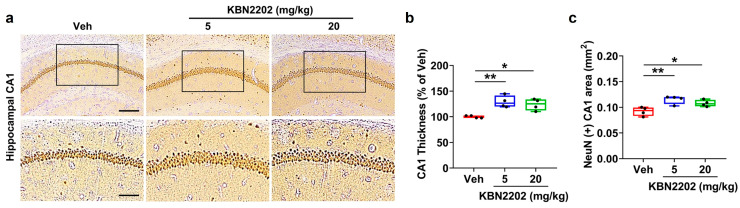
KBN2202 maintains neuronal structure in the hippocampal CA1 region of 5xFAD mice. (**a**) Representative DAB-stained images showing NeuN immunoreactivity in the CA1 region of vehicle (Veh)- and KBN2202-treated (5 or 20 mg/kg) 5xFAD mice. The lower panels represent magnified views of the boxed areas in the corresponding upper images. Scale bars: upper, 200 μm; lower, 100 μm. (**b**) The thickness of the stratum pyramidale was measured at three representative points, and the mean value was used to assess neuronal layer integrity. (**c**) Quantification of the NeuN-positive CA1 area (mm^2^). Quantification was performed in the CA1 region using paraffin-embedded coronal sections (*n* = 4 per group). Data are shown as individual points using box-and-whisker plots (median with minimum–maximum range). * *p* < 0.05 and ** *p* < 0.01 vs. Veh; one-way ANOVA followed by Dunnett’s post hoc test.

The hippocampal CA1 region is highly vulnerable to neurodegeneration in AD [25,26,27]. To assess whether KBN2202 could preserve neuronal integrity in this region, neuronal nuclei (NeuN) immunoreactivity was visualized using 3,3’-diaminobenzidine (DAB) staining, and the thickness of the stratum pyramidale was quantified. Representative NeuN-stained images are shown in Figure 1a. Quantitative analysis revealed that CA1 layer thickness was significantly increased in both the 5 mg/kg (129.3 ± 5.7%) and 20 mg/kg (123.9 ± 5.6%) KBN2202 groups compared with vehicle (Veh)-treated 5xFAD mice (100 ± 1.4%) (Figure 1b). Similarly, the NeuN-positive area within the CA1 region was significantly greater in both treatment groups (5 mg/kg, 0.11 ± 0.00 mm^2^; 20 mg/kg, 0.11 ± 0.00 mm^2^) relative to Veh controls (0.09 ± 0.00 mm^2^) (Figure 1c).

### 2.2. Preservation of Cortical Dendritic Architecture

**Figure 2:**
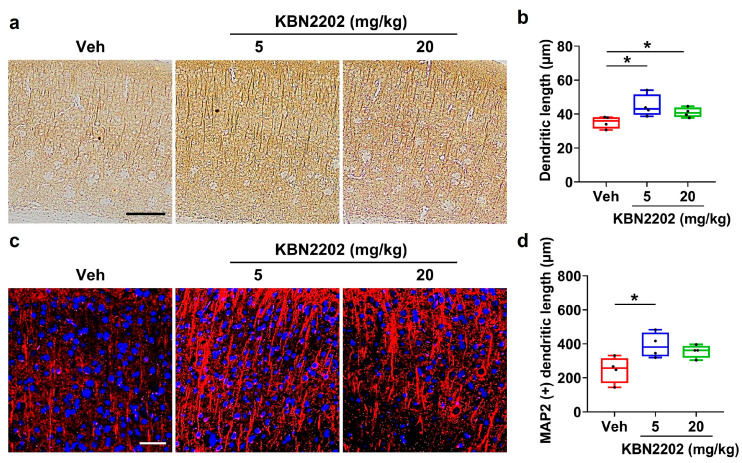
KBN2202 protects cortical dendritic architecture in 5xFAD mice. (**a**) Representative DAB-stained images of MAP2 immunoreactivity in the cortex. Scale bars: 200 μm. (**b**) Quantification of dendritic length from DAB-stained sections, showing significantly longer dendrites in both KBN2202-treated groups compared with vehicle (Veh) controls. (**c**) Representative fluorescence images of MAP2 immunostaining (red) in the cortex counterstained with DAPI (blue). Scale bars: 50 μm. (**d**) Quantification from fluorescence images showing a significant increase in the 5 mg/kg group, while the 20 mg/kg group exhibited a non-significant upward trend (*p* = 0.101 vs. Veh). Quantification was performed by measuring the mean dendritic length extending from cortical layer 5 in paraffin-embedded coronal sections obtained from each mouse (*n* = 4 per group). Data are shown as individual points using box-and-whisker plots (median with minimum–maximum range). * *p* < 0.05 vs. Veh; one-way ANOVA followed by Dunnett’s post hoc test.

Dendritic degeneration is a prominent pathological feature of AD and contributes to synaptic loss and cognitive decline [28,29]. To evaluate whether KBN2202 protects dendritic architecture in the cortex, microtubule-associated protein 2 (MAP2) immunoreactivity was assessed using both DAB and fluorescence staining. Representative MAP2-stained images are shown in Figure 2a,c. In the DAB-stained sections, cortical dendritic length was significantly increased in both the 5 mg/kg (44.8 ± 1.4 μm) and 20 mg/kg (41.1 ± 1.2 μm) KBN2202 groups compared with Veh-treated 5xFAD mice (34.4 ± 1.1 μm) (Figure 2b). In the fluorescence-stained sections, a significant increase in dendritic length was observed in the 5 mg/kg group (391.1 ± 22.1 μm), whereas the 20 mg/kg group (328.9 ± 16.5 μm, *p* = 0.101) showed a trend toward an increase relative to the Veh controls (249.4 ± 15.3 μm) (Figure 2d).

### 2.3. Enhancement of Adult Neurogenesis in the Subventricular Zone by KBN2202

**Figure 3:**
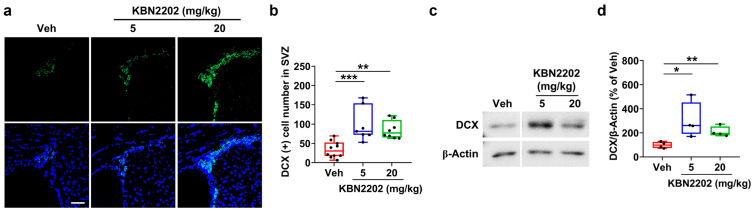
KBN2202 enhances neurogenesis in 5xFAD mice. (**a**) Representative immunofluorescence images of doublecortin (DCX)-positive cells (green) in the subventricular zone (SVZ) from vehicle (Veh, *n* = 10) and KBN2202-treated (5 or 20 mg/kg, *n* = 8 per group) mice, counterstained with DAPI (blue). Scale bars: 25 μm. (**b**) Quantification of DCX-positive cells in the SVZ was performed using coronal cryosections from both hemispheres of each mouse. Significant increases were observed in KBN2202-treated groups compared with Veh controls. (**c**) Representative immunoblot of DCX expression in brain lysates. (**d**) Densitometric quantification of DCX protein levels normalized to β-actin, showing significantly elevated expression in both 5 and 20 mg/kg KBN2202 groups (*n* = 4 mice per group) relative to Veh controls. Data are shown as individual points using box-and-whisker plots (median with minimum–maximum range). * *p* < 0.05, ** *p* < 0.01, and *** *p* < 0.001 vs. Veh; unpaired Student’s *t*-test or one-way ANOVA followed by Dunnett’s post hoc test.

Neurogenesis is markedly reduced in the subventricular zone (SVZ) of aged individuals and in AD, contributing to impaired brain plasticity and memory decline [30,31,32]. To evaluate whether KBN2202 could counteract this deficit, we assessed the expression of doublecortin (DCX), a marker of immature neurons. Representative images of DCX staining in the SVZ are shown in Figure 3a. Quantification revealed a significantly increased number of DCX-positive cells in KBN2202-treated mice (5 mg/kg, 98.6 ± 16.7 cells; 20 mg/kg, 85.0 ± 8.2 cells) compared with Veh-treated controls (33.7 ± 6.7 cells) (Figure 3b). Representative Western blot images are shown in Figure 3c. Consistently, Western blot analysis of brain lysates showed elevated DCX expression in both the 5 mg/kg (301.9 ± 74.4%) and 20 mg/kg (209.2 ± 21.4%) KBN2202-treated groups relative to Veh controls (100 ± 13.2%) (Figure 3d).

### 2.4. Effects of KBN2202 on Amyloidogenic Protein Expression

**Figure 4:**
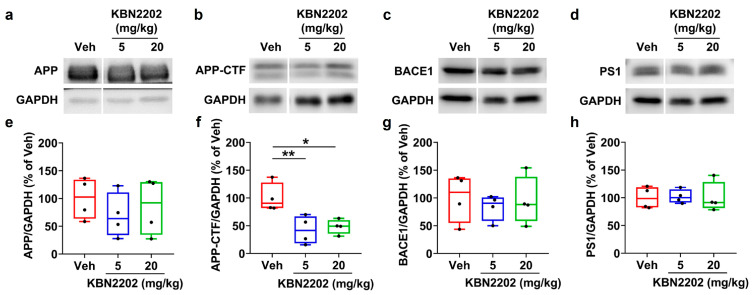
KBN2202 modulates amyloidogenic protein levels in the hippocampus of 5xFAD mice. (**a**–**d**) Representative immunoblots of full-length APP (**a**), APP C-terminal fragments (APP-CTFs) (**b**), BACE1 (**c**), and PS1 (**d**) in hippocampal lysates from vehicle (Veh)- and KBN2202-treated (5 or 20 mg/kg) mice. (**e**–**h**) Densitometric quantification of APP (**e**), APP-CTFs (**f**), BACE1 (**g**), and PS1 (**h**) protein levels normalized to GAPDH. APP-CTFs were reduced in both KBN2202-treated groups compared with Veh controls, whereas full-length APP, BACE1, and PS1 levels remained unchanged across groups. Data were obtained from three independent experiments using hippocampal samples (*n* = 4 per group). Data are shown as individual points using box-and-whisker plots (median with minimum–maximum range). * *p* < 0.05 and ** *p* < 0.01 vs. Veh; one-way ANOVA followed by Dunnett’s post hoc test.

The generation of Aβ peptides involves sequential cleavage of amyloid precursor protein (APP) by β-site APP cleaving enzyme 1 (BACE1) and γ-secretase (which contains presenilin-1 (PS1)), producing APP C-terminal fragments (APP-CTFs) as key intermediates in the amyloidogenic pathway [1,33]. To determine whether KBN2202 influences amyloidogenic processing, we analyzed the expression of full-length APP, APP-CTFs, BACE1, and PS1 in hippocampal lysates. Representative Western blot images are shown in Figure 4a–d, with corresponding densitometric quantifications in Figure 4e–h (APP, APP-CTFs, BACE1, and PS1, respectively). Quantitative analysis revealed a significant reduction in APP-CTFs in both KBN2202 treatment groups (5 mg/kg, 42.2 ± 12.7%; 20 mg/kg, 48.3 ± 6.6%) compared with Veh controls (100 ± 13.0%) (Figure 4f). In contrast, full-length APP (Figure 4e), BACE1 (Figure 4g), and PS1 (Figure 4h) did not differ significantly from those of Veh controls.

### 2.5. Reduction in Cortical and Hippocampal Aβ Plaque Burden by KBN2202

**Figure 5:**
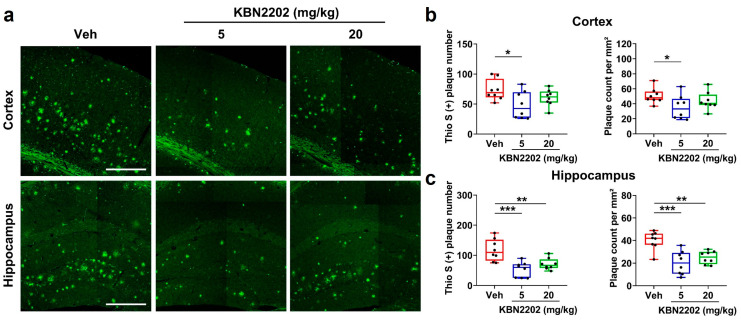

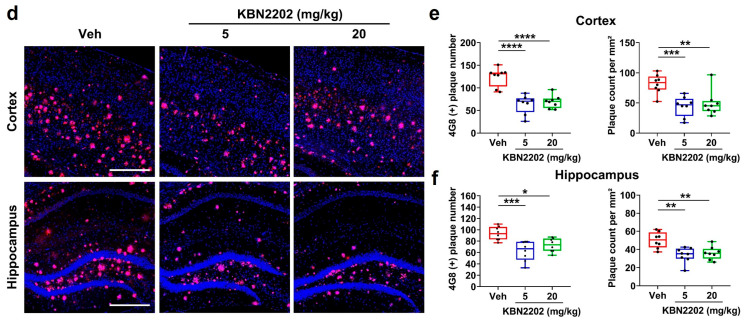
KBN2202 reduces Aβ plaque burden in the cortex and hippocampus of 5xFAD mice. (**a**) Representative thioflavin S (Thio S)-stained images (green) showing Aβ plaques in the cortex and hippocampus of vehicle (Veh)- and KBN2202-treated (5 or 20 mg/kg) mice. Scale bars: 400 μm. (**b**,**c**) Quantification of plaque number and count per mm^2^, demonstrating significant reductions in the cortex at 5 mg/kg (**b**) and in the hippocampus at both doses (**c**). (**d**) Representative 4G8 immunofluorescence images (red) of Aβ plaques counterstained with DAPI (blue). Scale bars: 400 μm. (**e**,**f**) Quantification of plaque number and count per mm^2^ showing significant decreases in both the cortex (**e**) and hippocampus (**f**) of KBN2202-treated groups compared with Veh controls. Quantification was performed on paraffin-embedded coronal sections from both hemispheres (*n* = 8 per group). Data are shown as individual points using box-and-whisker plots (median with minimum–maximum range). * *p* < 0.05, ** *p* < 0.01, *** *p* < 0.001, and **** *p* < 0.0001 vs. Veh; one-way ANOVA followed by Dunnett’s post hoc test.

Aβ plaque deposition is a pathological hallmark of AD and correlates with disease severity and cognitive decline [34]. Fibrillar Aβ plaques were visualized using thioflavin S (Thio S) staining, which selectively binds to β-sheet-rich amyloid aggregates to detect mature plaques [35]. Representative Thio S-stained images are shown in Figure 5a, with corresponding quantifications in Figure 5b,c. In the cortex, treatment with KBN2202 at 5 mg/kg led to a significant decrease in plaque number (48.3 ± 8.0 plaques) and plaque count per mm^2^ (35.0 ± 5.6 plaques/mm^2^), whereas the 20 mg/kg group (number: 60.5 ± 4.9 plaques; count per mm^2^: 43.8 ± 4.2 plaques/mm^2^) did not differ significantly from Veh-treated controls (number: 73.4 ± 6.1 plaques; count per mm^2^: 50.5 ± 3.6 plaques/mm^2^) (Figure 5b). In the hippocampus, both doses markedly reduced plaque number (Veh, 117.3 ± 12.8 plaques; 5 mg/kg, 53.0 ± 8.9 plaques; 20 mg/kg, 71.0 ± 6.7 plaques) and count per mm^2^ (Veh, 40.0 ± 2.9 plaques/mm^2^; 5 mg/kg, 20.2 ± 3.7 plaques/mm^2^; 20 mg/kg, 25.0 ± 2.0 plaques/mm^2^) (Figure 5c).

To further validate these findings, immunofluorescence staining was performed using the 4G8 antibody, which recognizes amino acids 17–24 of the Aβ sequence and detects both diffuse and compact plaques [36]. Representative images are shown in Figure 5d, with corresponding quantifications in Figure 5e,f. In the cortex, both doses attenuated amyloid pathology, showing smaller plaque numbers (5 mg/kg, 64.0 ± 7.3 plaques; 20 mg/kg, 69.3 ± 5.0 plaques) and lower plaque count per mm^2^ (5 mg/kg, 44.2 ± 5.9 plaques/mm^2^; 20 mg/kg, 49.2 ± 7.4 plaques/mm^2^) compared with Veh controls (number: 124.3 ± 7.3 plaques; count per mm^2^: 81.7 ± 5.6 plaques/mm^2^) (Figure 5e). In the hippocampus as well, both doses reduced plaque number (Veh, 93.4 ± 4.3 plaques; 5 mg/kg, 62.6 ± 6.1 plaques; 20 mg/kg, 72.8 ± 4.2 plaques) and a significant decrease in plaque count per mm^2^ was also observed (Veh, 50.2 ± 3.1 plaques/mm^2^; 5 mg/kg, 33.8 ± 2.9 plaques/mm^2^; 20 mg/kg, 36.0 ± 2.5 plaques/mm^2^) (Figure 5f).

### 2.6. Attenuation of Hippocampal Neuroinflammation and Amyloid Pathology by KBN2202

**Figure 6:**
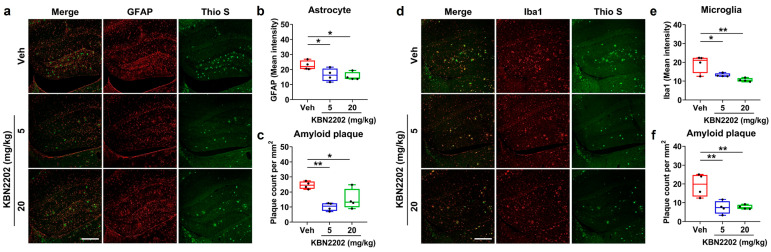
KBN2202 attenuates hippocampal neuroinflammation and amyloid pathology in 5xFAD mice. (**a**) Representative immunofluorescence images of GFAP-positive astrocytes (red) and thioflavin S (Thio S)-positive amyloid plaques (green) in the hippocampus of vehicle (Veh)- and KBN2202-treated (5 or 20 mg/kg) mice. Scale bars: 200 μm. (**b**,**c**) Quantification of GFAP immunoreactivity (**b**) and Thio S-positive plaque burden (**c**), demonstrating significant reductions in both KBN2202 groups compared with Veh controls. (**d**) Representative images of Iba1-positive microglia (red) and Thio S (green) staining in the hippocampus. Scale bars: 200 μm. (**e**,**f**) Quantification of Iba1 immunoreactivity (**e**) and Thio S-positive plaque burden (**f**), indicating decreased microglial activation and lower amyloid deposition after KBN2202 treatment. Quantification was performed using paraffin-embedded coronal sections (*n* = 4 per group). Data are shown as individual points using box-and-whisker plots (median with minimum–maximum range). * *p* < 0.05, ** *p* < 0.01 vs. Veh; one-way ANOVA followed by Dunnett’s post hoc test.

Neuroinflammation is a prominent pathological feature of AD, characterized by activation of astrocytes and microglia in regions with high amyloid burden [4,5,37]. To determine whether KBN2202 exerts anti-inflammatory effects, we evaluated astrocytic and microglial activation in the hippocampus of 5xFAD mice by immunofluorescence staining for glial fibrillary acidic protein (GFAP) and ionized calcium-binding adaptor molecule 1 (Iba1), respectively. Representative images are shown in Figure 6a,d. The fluorescence intensity of GFAP- and Iba1-positive cells was quantified independently, revealing marked reductions in both astrocytic and microglial activation in the treatment groups. Thio S co-staining with GFAP or Iba1 was performed to visualize the spatial association between amyloid plaques and glial activation, and each signal was quantified independently. KBN2202 treatment significantly reduced the intensity of GFAP-positive astrocytes (5 mg/kg, 16.4 ± 2.1% a.u.; 20 mg/kg, 15.4 ± 1.4 a.u.) (Figure 6b) and Iba1-positive microglia (5 mg/kg, 13.1 ± 0.5 a.u.; 20 mg/kg, 10.5 ± 0.5 a.u.) (Figure 6e) compared with Veh controls (GFAP: 22.8 ± 1.5 a.u.; Iba1: 19.2 ± 2.3 a.u.). Importantly, these decreases in gliosis were accompanied by a parallel reduction in amyloid plaque deposition, indicating that KBN2202 mitigates both neuroinflammation and amyloid pathology within affected hippocampal regions (GFAP + Thio S: Veh, 24.5 ± 1.2 plaques/mm^2^; 5 mg/kg, 10.1 ± 1.3 plaques/mm^2^; 20 mg/kg, 15.0 ± 3.4 plaques/mm^2^, Figure 6c) (Iba1 + Thio S: Veh, 19.3 ± 3.1 plaques/mm^2^; 5 mg/kg, 7.4 ± 1.7 plaques/mm^2^; 20 mg/kg, 7.6 ± 0.6 plaques/mm^2^, Figure 6f). Together, these findings show that KBN2202 suppresses glial activation and concomitantly reduces amyloid accumulation in the hippocampus of 5xFAD mice.

### 2.7. Improvement of Recognition Memory but Not Working Memory by KBN2202

**Figure 7:**
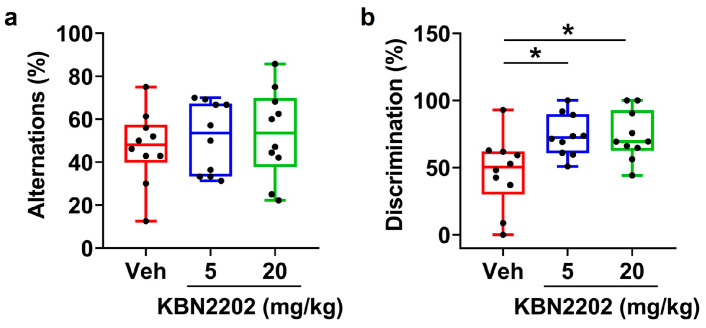
KBN2202 restores recognition memory but not working memory in aged 5xFAD mice. (**a**) Quantification of spontaneous alternation (%) in the Y-maze test. No significant differences were observed with either dose of KBN2202. (**b**) Quantification of the discrimination index (%) in the novel object recognition (NOR) test, showing a significant improvement in recognition memory in KBN2202-treated mice compared with vehicle (Veh) controls. Behavioral tests were performed with 10 mice per group. Data are shown as individual points using box-and-whisker plots (median with minimum–maximum range). * *p* < 0.05 vs. Veh; one-way ANOVA followed by Dunnett’s post hoc test.

Behavioral performance was evaluated to determine whether KBN2202 treatment could alleviate cognitive deficits in aged 5xFAD mice, which typically display impairments in both working and recognition memory at late disease stages [24]. Spatial working memory was assessed using the Y-maze spontaneous alternation test. Veh-treated 5xFAD mice showed reduced alternation performance (below 50%), consistent with previous findings in aged 5xFAD mice and reflecting the characteristic working memory deficits in this model [38,39]. KBN2202-treated groups did not show a statistically significant difference in alternation percentage compared with Veh controls (Figure 7a). In contrast, recognition memory was evaluated using the NOR test. Veh-treated 5xFAD mice (46.6 ± 8.5%) exhibited impaired discrimination between novel and familiar objects, whereas KBN2202-treated mice (5 mg/kg, 74.0 ± 4.9%; 20 mg/kg, 73.6 ± 5.8%) showed a significant improvement in the discrimination index, indicating recovery of recognition memory (Figure 7b). These results demonstrate that while spatial working memory remained unaffected, KBN2202 restored recognition memory, underscoring its ability to rescue memory processes dependent on cortical and hippocampal integrity.

## 3. Discussion

This study demonstrates that oral administration of KBN2202 confers multifaceted therapeutic benefits in 5xFAD mice. Importantly, these benefits were observed even when treatment was initiated at an advanced stage of AD pathology, a time point when therapeutic efficacy is typically diminished. Treatment began at nine months of age, when amyloid burden, synaptic degeneration, and gliosis are already extensive, reflecting the clinical reality that most patients initiate therapy only after substantial disease progression. Indeed, targeted therapies have historically shown limited efficacy when administered at late stages of AD [40,41].

In the present study, KBN2202 provided clear neuroprotection in key vulnerable regions of the AD brain. In the hippocampus, treatment increased the thickness of the CA1 stratum pyramidale, indicating preservation of pyramidal neuronal structure [26,27]. In the cortex, MAP2 staining revealed longer dendritic processes in KBN2202-treated mice, supporting its role in maintaining dendritic architecture [28]. Because structural integrity of pyramidal neurons and dendritic networks strongly correlates with cognitive performance, these findings highlight the compound’s ability to stabilize neuronal circuits despite progressive pathology [25]. Such stabilization may provide the structural foundation necessary for functional recovery, which could become evident with earlier initiation or extended therapeutic intervention.

Beyond structural preservation, KBN2202 induced an increase in DCX expression, a marker of neuronal precursor cells and immature neurons that reflects ongoing neurogenic activity [42]. Altered DCX expression has been reported in AD patients, particularly within the dentate gyrus, where residual DCX-positive cells may reflect an endogenous but insufficient attempt at neuronal replacement [10,43]. Impaired neurogenesis is increasingly recognized as a major contributor to reduced plasticity and adaptability in AD [30,32]. By increasing DCX expression, KBN2202 is expected to promote neurogenesis and may thereby counteract neurogenic deficits, which represent a hallmark of AD pathology—an outcome not addressed by current amyloid-targeting therapies such as monoclonal antibodies [11,44].

In this study, KBN2202 reduced APP-CTFs in the hippocampus at the molecular level and attenuated amyloid plaque deposition in both the cortex and hippocampus. Since APP-CTFs represent a critical intermediate in amyloidogenic processing, their reduction suggests upstream modulation of Aβ production, which could limit the accumulation of toxic oligomers implicated in synaptic dysfunction and neurotoxicity [1,33,45]. Previous studies have demonstrated that both fibrillar and diffuse plaques contribute to AD pathogenesis by triggering synaptic loss, inflammation, and neuronal injury, and that pharmacological interventions reducing these plaque types can delay disease progression [34,46]. By showing similar effects, KBN2202 appears capable of modulating amyloid pathology at both molecular and histological levels, thereby supporting its potential to attenuate AD progression.

KBN2202 lowered the levels of GFAP-positive astrocytes and Iba1-positive microglia in the hippocampus, indicating a reduction in neuroinflammation. This reduction in glial activation was accompanied by concurrent decreases in amyloid plaque deposition, suggesting that KBN2202 disrupts the feed-forward cycle linking amyloid accumulation and inflammatory responses [3,4]. Because chronic neuroinflammation exacerbates neuronal injury and impairs neurogenesis, its attenuation likely synergizes with KBN2202’s neuroprotective and regenerative actions [4,5,47].

In terms of behavioral outcomes, KBN2202 did not improve spatial working memory in the Y-maze, consistent with the notion that intervention at the symptomatic stage is insufficient to rescue short-term memory function [48,49]. In contrast, the compound significantly restored recognition memory in the NOR test. Recognition memory relies heavily on cortical and hippocampal integrity, regions where KBN2202 preserved neuronal and dendritic architecture, providing a mechanistic link between structural protection and functional recovery. These findings indicate that KBN2202 exerts selective benefits on recognition memory, even when working memory deficits remain irreversible at late disease stages.

KBN2202 has not yet been investigated for its direct molecular target; however, based on its chemical structure and pharmacological profile, it is likely to share mechanistic similarities with other salicylic acid-derived compounds. Such agents are known to exert pleiotropic effects through COX inhibition, NF-κB and AMPK modulation, and regulation of oxidative and inflammatory signaling, which collectively contribute to their anti-inflammatory, antioxidant, and neuroprotective properties [50,51]. These integrated actions may account for the broad therapeutic benefits of KBN2202 observed in this study. Interestingly, the biochemical and histological results did not show a clear dose dependence, as the 5 mg/kg treatment produced greater improvement than the 20 mg/kg dose. This non-linear response pattern may reflect the compound’s complex pharmacology, in which higher concentrations could trigger compensatory feedback mechanisms or reach a maximal effect, resulting in diminished efficacy. Further studies are warranted to identify the specific molecular target(s) of KBN2202 and to elucidate the mechanisms underlying this dose–response nonlinearity.

The current findings should be interpreted in light of several limitations. First, although KBN2202 elicited robust histopathological and molecular benefits, the precise molecular target of the compound remains to be elucidated. While reductions in APP-CTFs and plaque burden point toward modulation of amyloidogenic processing, it is unclear whether these effects reflect direct interactions with secretase complexes or indirect modulation of upstream cellular pathways. Mechanistic studies using genetic or pharmacological approaches will be needed to clarify its mechanism of action. Second, broader validation across different AD models, as well as translational studies in non-human primates or human-derived neuronal systems, will be required to establish the therapeutic relevance and safety profile of KBN2202. Third, this study was conducted using only male 5xFAD mice, which may limit the generalization of the findings across sexes. Biological sex differences in amyloid pathology, neuroinflammation, and neurogenesis have been reported in Alzheimer’s disease models, and hormonal or metabolic factors may influence drug responsiveness. Therefore, future studies should evaluate potential sex-dependent differences in drug response to determine whether the therapeutic efficacy of KBN2202 is consistently observed across both sexes. Finally, it will be important to investigate whether earlier or prolonged intervention can further enhance the cognitive benefits observed in the present study.

## 4. Materials and Methods

### 4.1. Animals and Housing Conditions

Male 5xFAD transgenic mice [B6.Cg-Tg(APPSwFlLon,PSEN1*M146L L286V*)6799Vas/Mmjax; JAX stock #034848] were obtained from The Jackson Laboratory (Bar Harbor, ME, USA) and housed in a pathogen-free facility under controlled environmental conditions (22 ± 2 °C, 50–60% humidity, 12/12 h normal light/dark cycle; lights on at 07:00 h). Mice were housed five per cage with wood-chip bedding and provided with food and water ad libitum. All experimental procedures were performed during the light phase in a quiet environment to minimize stress. Animals were gently handled and acclimated to the experimenter and testing room before all procedures. Every effort was made to minimize animal suffering and reduce the number of animals used in the study.

A total of 43 mice were used in the experiment: 15 in the Veh group and 14 in each of the KBN2202 5 mg/kg and 20 mg/kg groups. For behavioral testing, 10 animals were assigned to each group. Among them, four mice were used for Western blot analysis, another four for histological examination using paraffin-embedded sections, and the remaining two for pharmacokinetic analyses. In addition, a separate cohort of four mice per group (five in the Veh group) was processed for cryosectioning to examine neurogenesis in the SVZ. All animal procedures were approved by the Institutional Animal Care and Use Committee (IACUC) at K-MEDI Hub (approval numbers: KMEDI-23052203-00 and KMEDI-24061204-00) and conducted in accordance with the ARRIVE guidelines.

The same Veh-treated cohort used in our previous KBN2201 study (currently under revision) was included in the present analysis to ensure experimental consistency between studies. Quantitative data from the Veh group were shared between the two studies, whereas representative images were independently acquired for each experiment.

### 4.2. KBN2202 Treatment

At 9 months of age, corresponding to a symptomatic and advanced stage of AD-like pathology characterized by extensive amyloid deposition, gliosis, neuronal loss, and cognitive impairment [24,49], mice were randomly assigned to receive KBN2202 (5 or 20 mg/kg) or Veh. A 20 mg/mL stock solution (5% DMSO in phosphate-buffered saline (PBS)) of KBN2202 was diluted with PBS to final concentrations of 0.5 mg/mL and 2 mg/mL and administered at a volume of 10 µL/g body weight to achieve final doses of 5 mg/kg and 20 mg/kg, respectively. The compound was administered once daily by oral gavage in the morning for 12 weeks. Drug administration was continued during the behavioral testing period following the 3-month treatment and maintained until the completion of the experiment.

### 4.3. Behavioral Assessment 

Behavioral tests were performed during the light phase (09:00–16:00) in a quiet, dimly lit room under consistent environmental conditions. Mice were acclimated to the testing room for at least 30 min before each session to minimize stress and variability. All animals were behaviorally naïve and had no prior exposure to any behavioral apparatus before testing. Each behavioral task (Y-maze and NOR) was conducted once per mouse following the 12-week treatment period, and all tests were performed in a single session for each animal.

#### 4.3.1. Y-Maze 

Spatial working memory was assessed using the Y-maze spontaneous alternation test. The apparatus consisted of three arms (40 cm long × 15 cm high) arranged at 120° angles. Each mouse was placed at the end of one arm and allowed to explore freely for 5 min. Arm entries were recorded by a video camera and analyzed with EthoVision XT (version 17, Noldus, Wageningen, The Netherlands). Spontaneous alternation was defined as consecutive entries into all three arms without repetition, and the alternation (%) was calculated as = [number of alternations / (total arm entries – 2)] × 100.

#### 4.3.2. NOR

Recognition memory was assessed using the NOR test. Mice were first habituated to an open-field arena (40 cm × 40 cm × 40 cm) for 10 min. On the following day, two identical objects were placed in the arena for a 10 min training session. After a 24-h delay, one familiar object was replaced with a novel object, and mice were allowed to explore freely for 5 min. Exploration time was manually recorded, and the discrimination (%) was calculated as (time spent exploring the novel object / total exploration time) × 100.

### 4.4. Tissue Preparation and Immunohistochemistry 

#### 4.4.1. Tissue Preparation

After completion of the behavioral tests, animals were anesthetized with 2–3% isoflurane (Hana Pharm, Seoul, Republic of Korea) and transcardially perfused with phosphate-buffered saline (PBS, pH 7.4), followed by fixation with 4% paraformaldehyde (PFA) for histological analysis. Brains were post-fixed and processed for either paraffin embedding or cryosectioning. For paraffin sections, tissues were dehydrated, embedded in paraffin, and coronally sectioned at 4 µm thickness. For cryosections, brains were cryoprotected in 30% sucrose, embedded in optimal cutting temperature (OCT) compound, and coronally sectioned at 20 µm thickness. Paraffin sections underwent antigen retrieval in citrate buffer (Sigma-Aldrich, C8532, St. Louis, MO, USA), whereas cryosections were processed directly.

#### 4.4.2. DAB Staining

For DAB staining, paraffin-embedded brain sections were deparaffinized, rehydrated, and treated with 0.3% hydrogen peroxide in distilled water for 15 min to block endogenous peroxidase activity. After rinsing with PBS and blocking with 5% normal serum for 1 h at room temperature, sections were incubated with primary antibodies overnight at 4 °C, followed by a horseradish peroxidase (HRP)-conjugated secondary antibody for 1 h. Immunoreactivity was visualized using a DAB substrate kit (Vector Laboratories, PK-6100, Newark, CA, USA), and sections were rinsed, dehydrated, and coverslipped for microscopic analysis.

#### 4.4.3. Immunohistochemistry

Aβ pathology was evaluated using Thio S (Sigma-Aldrich, T1892) staining for fibrillar plaques and immunofluorescence labeling with the 4G8 anti-Aβ antibody (mouse monoclonal, 1:200, BioLegend, 800701, San Diego, CA, USA) for total deposits. Additional markers included NeuN (mouse monoclonal, 1:200, Cell Signaling Technology, 24307, Danvers, MA, USA) and MAP2 (mouse monoclonal, 1:200, Merck, MAB3418, Darmstadt, Germany) for neuronal integrity; GFAP (mouse monoclonal, 1:200, Sigma-Aldrich, G3893) and Iba1 (rabbit polyclonal, 1:200, Wako, 019-19741, Osaka, Japan) for astrocytic and microglial activation; and DCX (rabbit polyclonal, 1:200, Abcam, ab18723, Cambridge, UK) for neurogenesis.

Immunoreactivity was detected with FITC- and Cy3-conjugated secondary antibodies: anti-mouse IgG (goat, Cy3-conjugated, 1:200; Thermo Fisher Scientific, Waltham, MA, USA) and anti-rabbit IgG (goat, Cy3- or FITC-conjugated, 1:200; Thermo Fisher Scientific). All sections were counterstained with DAPI (4’,6-diamidino-2-phenylindole, Sigma, D9542) and mounted with antifade medium (Biomeda, M01, Foster City, CA, USA).

#### 4.4.4. Image Acquisition

Fluorescence images were acquired with a Zeiss LSM900 confocal microscope (Carl Zeiss, Oberkochen, Germany), and bright-field images were captured with an Olympus microscope (Tokyo, Japan). Imaging parameters were kept constant across all experimental groups. Quantitative analyses were performed using ImageJ (version 1.52a, NIH, Bethesda, MD, USA) and Icy software (version 2.1.0.0, Institut Pasteur, Paris, France).

### 4.5. Protein Extraction and Western Blot Analysis

#### 4.5.1. Protein Extraction

Brain tissues were rapidly dissected on ice and homogenized in ice-cold radioimmunoprecipitation assay (RIPA) lysis buffer supplemented with protease and phosphatase inhibitors. Homogenates were incubated on ice for 30 min with intermittent vortexing and then clarified by centrifugation at 14,000× *g* for 1 h at 4 ℃. The supernatant was collected, and protein concentrations were determined using the Bradford assay (Bio-Rad, #5000205, Hercules, CA, USA). Protein samples were aliquoted in equal volumes and stored at −80 °C until use for immunoblotting.

#### 4.5.2. Western Blot Analysis

Equal amounts of protein (20–30 µg) were separated by sodium dodecyl sulfate–polyacrylamide gel electrophoresis (SDS–PAGE) and transferred to polyvinylidene difluoride (PVDF) or nitrocellulose (NC) membranes. Membranes were blocked in 3% bovine serum albumin (BSA) and incubated overnight at 4 °C with primary antibodies against full-length APP (rabbit monoclonal, 1:2000, Cell Signaling Technology, #29765), APP-CTFs (rabbit polyclonal, 1:1000, Sigma-Aldrich, A8717), BACE1 (rabbit monoclonal, 1:1000, Abcam, ab183612), PS1 (rabbit monoclonal, 1:1000, Cell Signaling Technology, 5643), DCX (rabbit polyclonal, 1:1000, Abcam, ab18723) and β-actin or glyceraldehyde 3-phosphate dehydrogenase (GAPDH) (mouse monoclonal, 1:2000, Santa Cruz Biotechnology, sc-47778 or sc-32233, Dallas, TX, USA).

#### 4.5.3. Densitometric Quantification

After incubation with HRP-conjugated secondary antibodies, bands were visualized using enhanced chemiluminescence (ECL) reagents (Cytiva, RPN2106, Marlborough, MA, USA) and imaged with a Fusion Solo S chemiluminescence imaging system (Vilber, Collégien, France). Band intensities were quantified using ImageJ software (version 1.52a, NIH, Bethesda, MD, USA) and normalized to the corresponding β-actin or GAPDH signals to control for loading variability.

### 4.6. Statistical Analysis

Data are expressed as mean ± standard error of the mean (SEM). Normality of data distribution was assessed using the Shapiro–Wilk test prior to statistical testing. When the data followed a normal distribution, group comparisons were performed using one-way ANOVA followed by Dunnett’s post hoc test where appropriate. For non-normally distributed data, the Kruskal–Wallis test was applied. All comparisons between study groups were considered. Statistical analyses and figure generation were performed in GraphPad Prism 10 (GraphPad Software, San Diego, CA, USA), and differences were considered statistically significant at *p* < 0.05.

## 5. Conclusions

In summary, KBN2202 demonstrated multifaceted neuroprotective actions in a severe model of AD, including preservation of hippocampal and cortical neuronal architecture, enhancement of adult neurogenesis, attenuation of amyloidogenic processing and plaque deposition, and reduction in neuroinflammation. In addition, KBN2202 restored recognition memory in the NOR test, although spatial working memory deficits persisted. The convergence of structural, regenerative, molecular, and cognitive benefits underscores the potential of KBN2202 as a multitarget therapeutic candidate. These findings warrant further evaluation of KBN2202 in preclinical studies initiated at earlier disease stages and, ultimately, in clinical trials to assess its potential as a disease-modifying therapy for AD.

## Data Availability

The datasets generated and analyzed in this study are available from the corresponding author upon reasonable request.

## Abbreviations

The following abbreviations are used in this manuscript:

Aβ	Amyloid-β
AD	Alzheimer’s disease
APP	Amyloid precursor protein
APP-CTFs	Amyloid precursor protein C-terminal fragments
BACE1	β-site APP cleaving enzyme 1
BSA	Bovine serum albumin
DAB	3,3’-Diaminobenzidine
DAPI	4’,6-diamidino-2-phenylindole
ECL	Enhanced chemiluminescence
GAPDH	Glyceraldehyde 3-phosphate dehydrogenase
GFAP	Glial fibrillary acidic protein
HRP	Horseradish peroxidase
Iba1	Ionized calcium-binding adaptor molecule 1
LPS	Lipopolysaccharide
MAP2	Microtubule-associated protein 2
MPTP	1-methyl-4-phenyl-1,2,3,6-tetrahydropyridine
NC	Nitrocellulose
NOR	Novel object recognition
OCT	Optimal cutting temperature
PBS	Phosphate-buffered saline
PFA	Paraformaldehyde
PS1	Presenilin-1
PVDF	Polyvinylidene difluoride
RIPA	Radio immuno precipitation assay
SDS-PAGE	Sodium dodecyl sulfate-polyacrylamide
Thio S	Thioflavin S
